# MicroRNA expression and gene regulation drive breast cancer progression and metastasis in PyMT mice

**DOI:** 10.1186/s13058-016-0735-z

**Published:** 2016-07-22

**Authors:** Ruben Nogales-Cadenas, Ying Cai, Jhih-Rong Lin, Quanwei Zhang, Wen Zhang, Cristina Montagna, Zhengdong D. Zhang

**Affiliations:** 1 Department of Genetics, Albert Einstein College of Medicine, Bronx, NY 10461 USA; 2 Department of Pathology, Albert Einstein College of Medicine, Bronx, NY 10461 USA; 3 Albert Einstein College of Medicine, Michael F. Price Center, 1301 Morris Park Avenue, Room 353A, Bronx, NY 10461 USA

**Keywords:** PyMT mouse model, Breast cancer, MicroRNA, Cancer progression, Metastasis, Regulatory modules

## Abstract

**Background:**

MicroRNAs (miRNAs) are small non-coding RNA molecules of about 22 nucleotides which function to silence the expression of their target genes. Numerous studies have shown that miRNAs are not only key regulators in important cellular processes but are also drivers in the development of many diseases, especially cancer. Estrogen receptor positive luminal B is the second most common but the least studied subtype of breast cancer. Only a few studies have examined the expression profiles of miRNAs in luminal B breast cancer, and their regulatory roles in cancer progression have yet to be investigated.

**Methods:**

In this study, using polyoma middle T antigen (PyMT) mice, a widely used luminal B breast cancer model, we profiled microRNA (miRNA) expression at four time points that represent different key developmental stages of cancer progression. We considered the expression of both miRNAs and messenger RNAs (mRNAs) at these time points to improve the identification of regulatory targets of miRNAs. By combining gene functional and pathway annotation with miRNA-mRNA interactions, we created a PyMT-specific tripartite miRNA-mRNA-pathway network and identified novel functional regulatory programs (FRPs).

**Results:**

We identified 151 differentially expressed miRNAs with a strict dual nature of either upregulation or downregulation during the whole course of disease progression. Among 82 newly discovered breast-cancer-related miRNAs, 35 can potentially regulate 271 protein-coding genes based on their sequence complementarity and expression profiles. We also identified miRNA-mRNA regulatory modules driving specific cancer-related biological processes.

**Conclusions:**

In this study we profiled the expression of miRNAs during breast cancer progression in the PyMT mouse model. By integrating miRNA and mRNA expression profiles, we identified differentially expressed miRNAs and their target genes involved in several hallmarks of cancer. We applied a novel clustering method to an annotated miRNA-mRNA regulatory network and identified network modules involved in specific cancer-related biological processes.

**Electronic supplementary material:**

The online version of this article (doi:10.1186/s13058-016-0735-z) contains supplementary material, which is available to authorized users.

## Background

Breast cancer is one of the most common types of cancer among American women - about 12 % of women in the USA will develop invasive breast cancer during their lifetime - and it is a leading cause of cancer death. It is a heterogeneous disease, with substantial genotypic and phenotypic diversity [1]. Depending on the expression status of estrogen receptor (ER), progesterone receptor (PR), and human epidermal growth factor receptor 2 (HER2), it can be classified into four molecular subtypes: luminal A, luminal B, HER2-positive, and basal-like (or triple-negative) breast cancer. In a widely used mouse model of breast cancer, mammary gland-specific expression of the polyoma middle T (PyMT) oncoprotein under the control of the MMTV promoter/enhancer in transgenic mice (MMTV-PyMT) results in widespread transformation of the mammary epithelium and subsequent development of multifocal mammary adenocarcinomas and metastatic lesions in the lymph nodes and in the lungs [2]. Breast cancer in PyMT mice is particularly noted by its short latency, high penetrance, and a high incidence of lung metastasis [3]. Tumor formation and progression in these mice can be divided into four stages: hyperplasia, adenoma/mammary intra-epithelial neoplasia, early carcinoma, and late carcinoma [4] (we refer to all these four stages as tumor in this study). Whole-genome array profiling indicates that PyMT tumors most closely resemble the luminal B subtype of human breast cancer [5], although end-stage PyMT tumors are ER-negative and PR-negative [4]. Most genetically engineered mouse models that target oncogenes such as PyMT do not fully recapitulate several aspects of the development of human breast cancer. Not only are transgenes expressed at a level different from that of the same oncogenes in human breast cancer, but also throughout the ductal tree [6] they do not target the cell types that are the cells of origin in human breast cancer. Despite these limitations, the use of the PyMT mouse model and other similarly engineered models has been instrumental in elucidating the genetics and biology of breast cancer.

In addition to numerous protein-coding genes, many microRNAs (miRNAs) also play important roles in breast cancer. Since their discovery over two decades ago, miRNAs have been recognized as important regulators of many key cellular processes including development [7], cell cycle progression [8], differentiation [9], and apoptosis [10]. Their dysregulation occurs in various types of cancer [11] and is associated with different stages and aspects such as tumor initiation, drug resistance, and metastatic spread of the disease [12]. While some miRNAs have similar expression patterns across all cancer types, others are cell-type-specific and thus could potentially serve as cancer biomarkers [13]. A recent study using microarray and machine learning data analysis identified a small number of miRNAs differentially expressed in luminal B breast cancer [14]. Another study of miRNA expression in different breast cancer subtypes also identified positive and negative miRNA signatures for ER-positive, PR-positive, and Her2-positive luminal B tumors [15]. From carcinogenesis to metastasis, like any other types of cancer, the development of breast cancer is a multistage process. MiRNAs have been associated with different developmental stages including epithelial to mesenchymal transition (EMT), migration, invasion, and angiogenesis of breast cancer [16].

Affecting approximately 20 % of all patients with breast cancer, luminal B is the second most common but the least studied subtype of breast cancer. Only a few studies have examined the expression profiles of miRNAs in luminal B breast cancer [17–21], and the regulatory roles of miRNAs in the progression of the disease have yet to be investigated. In this study, using a luminal B breast cancer mouse model we profiled miRNA expression at four time points that represent different key stages of cancer progression and identified miRNAs differentially expressed between tumor and normal mammary gland cells. We considered the expression of both miRNAs and messenger RNAs (mRNAs) at multiple time points to improve the identification of potential targets of miRNAs. By combining gene functional and pathway annotation with miRNA-mRNA interactions, we created a PyMT-specific tripartite miRNA-mRNA-pathway network and identified novel functional regulatory programs (FRPs), that is miRNA-mRNA regulatory modules relevant to the development of luminal B breast cancer. The identification of novel cancer-related miRNAs and the FRPs shed new light on the disease mechanisms behind breast cancers, and will help the development of new biomarkers for early cancer detection and drug targets for plausible treatments.

## Methods

### PyMT samples

F1 female mice (PyMT mice hereafter) heterozygous for the PyMT transgene were obtained by random breeding of male PyMT mice (FVB/N-Tg(MMTV-PyVT) 634Mul/J mice, Stock Number: 002374, the Jackson Laboratory) with homozygous FVB female mice. After the mice developed breast cancer, they were used as tumor cases, while homozygous FVB female mice were used as controls. Four time points representing progression to malignancy in the PyMT mouse breast cancer model were sampled: hyperplasia, adenoma/mammary intraepithelial neoplasia (MIN), early carcinoma, and late carcinoma with lung metastasis at weeks 6, 8, 10, and 12 respectively [22]. At each time point, three PyMT mice and three age-matched controls were sacrificed; mammary tumors and normal mammary glands were collected, snap-frozen and kept at −80 °C. Thus, in this study we analyzed 24 samples in total, with three tumor samples as cases and three normal samples as controls at each of four time points. All tumor samples were from primary tumors of mammary glands.

All tumor samples from PyMT mice at 6, 8, 10, and 12 weeks of age were examined and confirmed by a pathologist. Typical morphologic features of those different tumor stages were observed (Additional file 1: Figure S1). As expected, tissues from the hyperplasia and adenoma/MIN stages contain normal mammary gland cells. In carcinoma samples, fibroblast cells can be a major concern. We estimated the sample purity by the percentage of infiltrating cells. Based on H&E staining, the pathology report described less than 5 % stromal and muscle cells and very few inflammatory cells. In general, we had greater than 90 % tumor cells in our carcinoma samples, which surpassed The Cancer Genome Atlas (TCGA) standard (over 60 % tumor cells for human tumor samples). Unlike human samples, tumor samples from the PyMT mice used in this study were filled with tumor epithelial cells and there were very few infiltrating cells due to the acute aggressiveness of tumors in PyMT mice.

### Small RNA extraction and sequencing

Total RNAs and non-coding RNAs (ncRNAs) were extracted from frozen samples using the miRNeasy mini kit (Qiagen, Valencia, CA, USA) according to the manufacturer's protocol. The Agilent 2100 Bioanalyzer was used to check RNA quality. Total RNA was used to create small RNA libraries using the Illumina TruSeq Small RNA Library Preparation Kit (version 1). Libraries were prepared according to the manufacturer's instructions. Purified libraries were used to sequence on Hiseq2500 single-end 1 × 50 b read-length according to standard protocols.

### Small RNA-sequencing (RNA-seq) and RNA-seq data analysis

Using a custom-built analysis pipeline, we processed small RNA-seq data in three steps - adaptor/tag removal, genomic alignment, and comparison with the miRBase [23] - to identify transcribed miRNAs and measure their expression levels. In the first step of data preprocessing, extraneous sequences - the barcode tag (used for sample multiplexing) and the Illumina adaptor - were removed from the 5' and 3' ends, respectively, of each read. After this trimming step, reads 17 to 27 bases long (the size range of mature miRNAs) were aligned to the mouse reference genome assembly (mm10) and RefSeq (release 61) using Bowtie. The locations of read-to-genome alignments were compared with those of known miRNA genes from miRBase. The number of reads that overlap more than 50 % of an miRBase-defined miRNA was counted and used as the expression level of this miRNA. We used edgeR to identify miRNAs expressed differentially between tumor and normal samples [24] (Additional file 2: Table S1).

For RNA-seq data, a separate analysis pipeline was designed and implemented. Sequence reads in FASTQ files were first trimmed for adapter sequences using quart [25]. They were aligned to the mouse reference genome assembly (mm10) using GSNAP [26, 27], which detects novel splicing events and known splice junctions based on the ENSEMBL mouse gene annotations [26], and then assigned to genes using HTSeq-count, a component of the HTSeq Python library [28]. Assignments were made using the union strategy, and alignments with a quality score lower than 10 were excluded. Genes differentially expressed between tumor and normal samples were identified using DESeq [29] (Additional file 2: Table S2). Finally, differentially expressed miRNAs were compared with the ones included in the miRCancer database [30] to identify similar expression in breast cancer and other cancer types.

### Computational miRNA targets prediction

We used the miRNA-mRNA interactions in the M3RNA Database derived from integration of predicted and experimentally validated interactions from different databases [31]. From this database we gathered 1,763,884 potential interactions between 834 miRNAs and 19,876 mRNAs for the whole mouse genome. We tested the correlation between miRNA and mRNA expression to further reduce the false positives in their predicted interactions. We first used a quantile-based method to normalize the counts of small RNA-seq reads from the 24 tumor and control samples and then computed the Spearman correlation for each pair of miRNA and mRNA predicted to interact by base pairing. All interactions with non-negative correlation were filtered out.

**Fig. 1:**
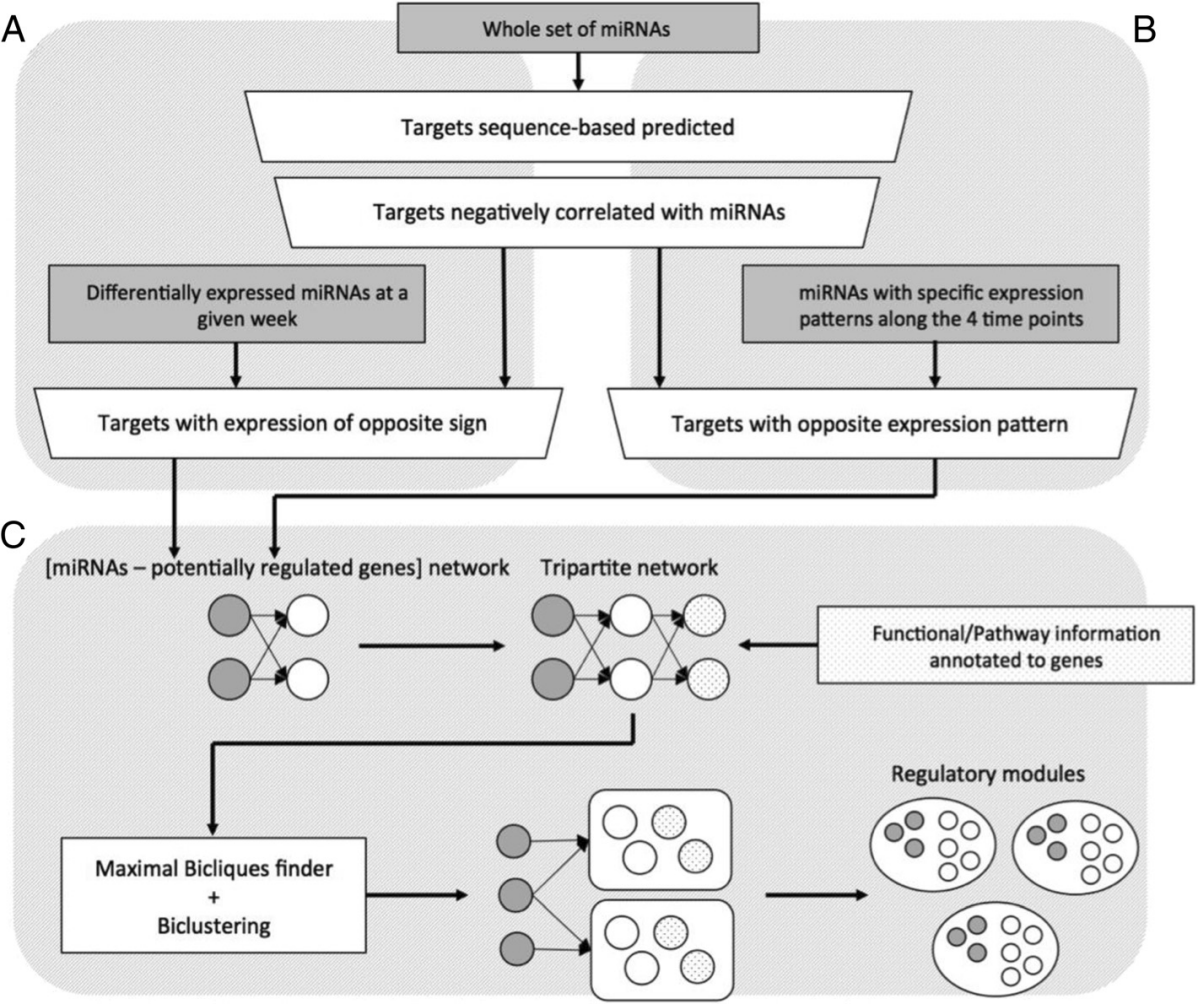
MicroRNA (miRNA) sequencing data analysis. **a** Identification of stage-specific differentially expressed miRNA-messenger RNA (mRNA) regulatory network. At each time point, for a differentially expressed miRNA and one of its mRNA targets predicted based on their sequence complementarity from the m3RNA database, we calculated the correlation between their expression levels (reads per kilobase million (RPKM)) from all 24 samples. Any miRNA-mRNA interaction with positive correlation was filtered out. Thus, only miRNA-mRNA pairs with opposite differential expression were included in the regulatory network for each cancer developmental stage. **b** Identification of overall transition pattern-specific miRNA-mRNA regulatory network. We classified miRNAs into 27 groups based on the overall expression patterns of the three consecutive stage transitions. In each group, only mRNA-miRNA pairs with opposite transition patterns were considered. **c** Identification of miRNA regulatory modules. We first annotated genes in a miRNA-mRNA regulatory network with functional and structural terms from Gene Ontology Biological Process, Kyoto Encyclopedia of genes and genomes (KEGG) pathways, Panther pathways, and Interpro domains. Using a maximal biclique analysis followed by bi-clustering, we then identified sets of coherently related genes and annotation terms. The miRNA regulatory modules were formed by adding miRNAs to corresponding sets that they potentially regulate

To construct miRNA-mRNA bipartite networks we considered four time points in two different ways, either independently or together through transitions (Fig. 1a and b). In the first approach, at each time point for each miRNA, only those interactions with potential targets having ectopic expression levels of opposite sign remained. In the second approach, the transition for each miRNA is discretized into three classes. With this aim, transitions (*T*) in miRNA pseudo fold-change (*ϕ*FCs) expression (tumor samples over normal samples) between two consecutive time points (*t* + 1 and *t*) were calculated:

$$ {T}_i=\phi F{C_i}^{\left(t+1\right)}\hbox{--} \phi F{C_i}^{(t)} $$

when *ϕ*FC of miRNA *i* at a given time point *t* was calculated by

$$ \phi F{C}_i^{(t)}\kern0.5em =\kern0.5em \left\{{}_{-{2}^{- \log F{C}_i^{(t)}\kern4em }\mathrm{if}\kern0.5em  \log F{C}_i^{(t)}\kern0.5em <\kern0.5em 0}^{2^{\log F{C}_i^{(t)}}\kern5em \mathrm{if}\kern0.5em  \log F{C}_i^{(t)}\kern0.5em \ge \kern0.5em 0}\right.. $$

**Fig. 2:**
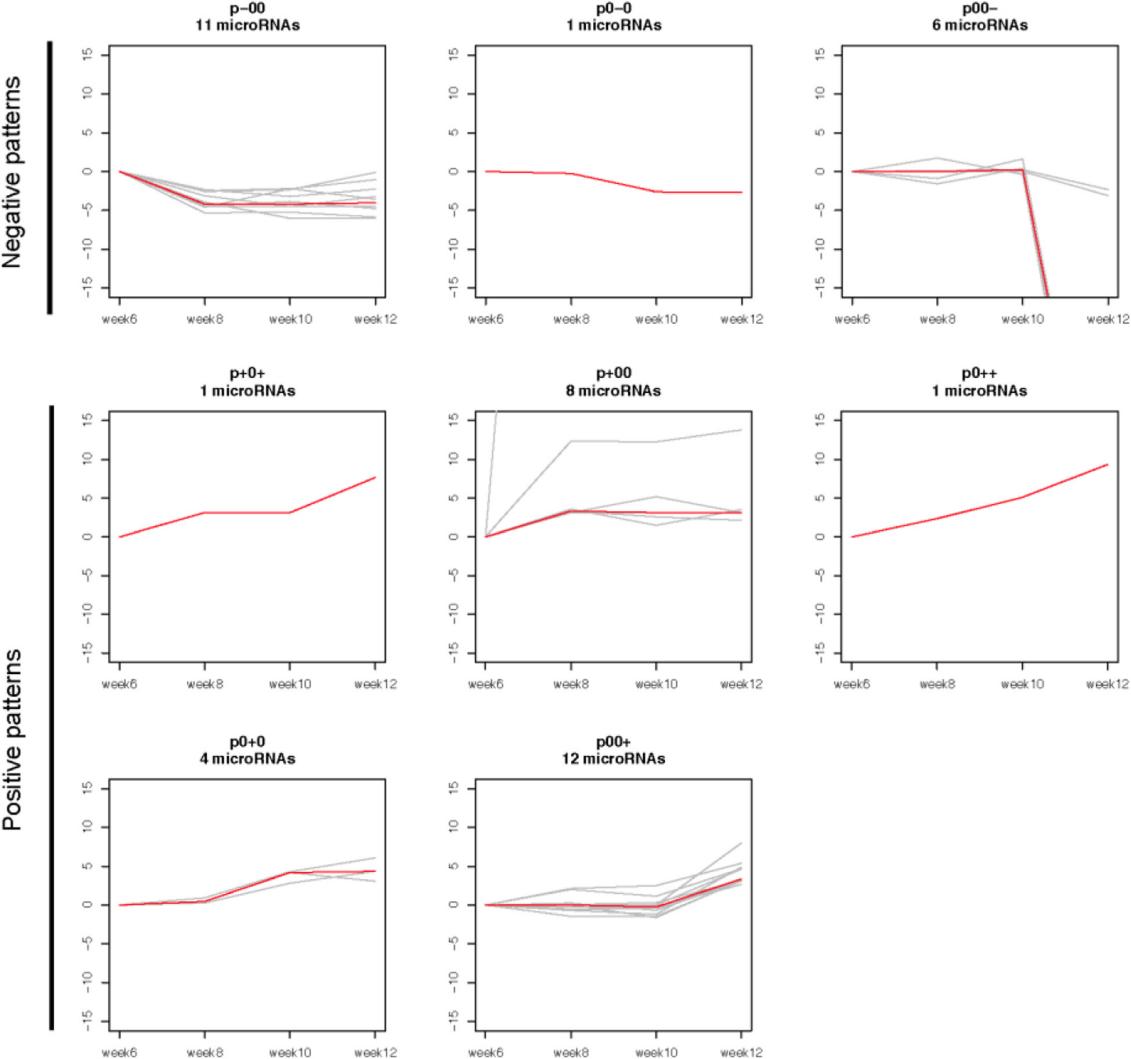
Transition patterns of microRNA (*miRNA*) expression during cancer progression. Each *gray line*, re-based to 0 at week 6, shows the pseudo fold-changes (*ϕ*FCs) of a particular miRNA at the four assayed time points. Expression change during each stage transition was discretized into increase (+), no change (*0*), or decrease (–). All miRNAs with the same transition pattern, e.g., *p–00*, are plotted together in one plot, in which the transition pattern and the number of miRNAs are given at the *top*. The *red line* in each plot indicates the median *ϕ*FCs. Only positive and negative patterns without opposite changes are shown here. See Additional file 1: Figure S2 for the whole set of 27 transition patterns

Based on their mean *μ* and standard deviation *σ*, they are discretized into three classes: increase, *C*_*i*_ = ' + ' if *T*_*i*_ > *μ* + 0.4 × *σ*; no change, *C*_*i*_ = '0' if *μ* – 0.4 × *σ < T*_*i*_ < *μ* + 0.4 × *σ*; and decrease, *C*_*i*_ = '–' if *T*_*i*_ < *μ* – 0.4 × *σ*. Because there are three classes for each of the three transitions between four consecutive time points, there are 3^3^ = 27 different transition patterns: '+++', '++0', '++ − ', and so on. In our downstream analysis, we focused on miRNAs with a non-increase (involving only '0' or ' + ' transitions) or non-decrease (involving only '0' or '–' transitions) trend in their expression. miRNAs and their potential targets with corresponding opposite transition patterns (Fig. 2) were used to build the interaction networks, which were later integrated with functional annotations and pathways for identification of regulatory modules.

After this filtering process, we compared the potential interacting miRNAs-mRNAs pairs with those reported in the CancerMiner study by considering the recurrent score computed for all types of cancer, the specific score computed for breast cancers and the minimum score computed for any of the 11 human cancer types [32].

### Functional and pathway enrichment analysis

Pairs of miRNAs and their targeted genes were analyzed for enrichment of biological functions. Using GeneCodis [33, 34], we examined Gene Ontology Biological Process terms [35], Kyoto Encyclopedia of Genes and Genomes (KEGG) pathways [36], and Panther Pathways [37] annotations. We used the singular enrichment analysis functionality of the tool and the hypergeometric statistical test to calculate the *P* values with multi-test correction by the false discovery rate (FDR) method. As the reference background for the statistical test, we used the whole mouse gene set from Ensembl (Release 65).

Enriched terms were manually annotated based on the 10 different hallmarks of cancer proposed by Hanahan and Weinberg [38]. We considered an additional category 'neural genetics', as some of the terms seemed to fit in this description, in agreement with studies relating both cancer genetics and neuronal disorders such as Alzheimer's disease [39]. See Additional file 2: Table S3 for correspondence between enriched terms and categories.

### Identification of functional-regulatory programs

Due to the high density of the networks, a simple approach such as clustering could not provide good results. Instead, given the functional relatedness of the miRNAs and mRNAs from a single module, for each network, we annotated the mRNAs from each module with Gene Ontology Biological Process terms, Interpro motifs [40], KEGG and Panther Pathways and thus extended bipartite networks into tripartite networks (Fig. 1c). Subnetworks including only mRNAs and their biological terms were then searched for statistically significant maximal bicliques (MBs) or, equivalently, the closed item sets [41]. As GeneCodis enrichment analysis is based on the identification of closed item sets from annotated genes, we used it to identify the MBs from the annotated mRNAs. Each MB is a subset of mRNAs and biological terms. In our study, we only considered MBs that are statistically significant compared with the whole mouse gene set and used a biclustering-based method [42] to remove the redundancy of overlapping genes and biological terms in the initial MBs. We considered in each case MBs including the minimum number of genes that maximize the general silhouette value, guaranteeing less overlap with other MBs and the best inner coherence. For each week, respectively we used 8, 7, 7, and 6 as minimum support. We also considered the miRNAs with positive and negative trending patterns along the 4 weeks, in both cases we used a minimum support of 4. To reduce false positives, only MBs with positive silhouette values were further considered. Finally, we constructed the final FRPs by adding to each MB the miRNAs connected with the mRNAs included in that MB.

## Results and discussion

### Global miRNA expression profiles and potential targets in PyMT mice

**Fig. 3:**
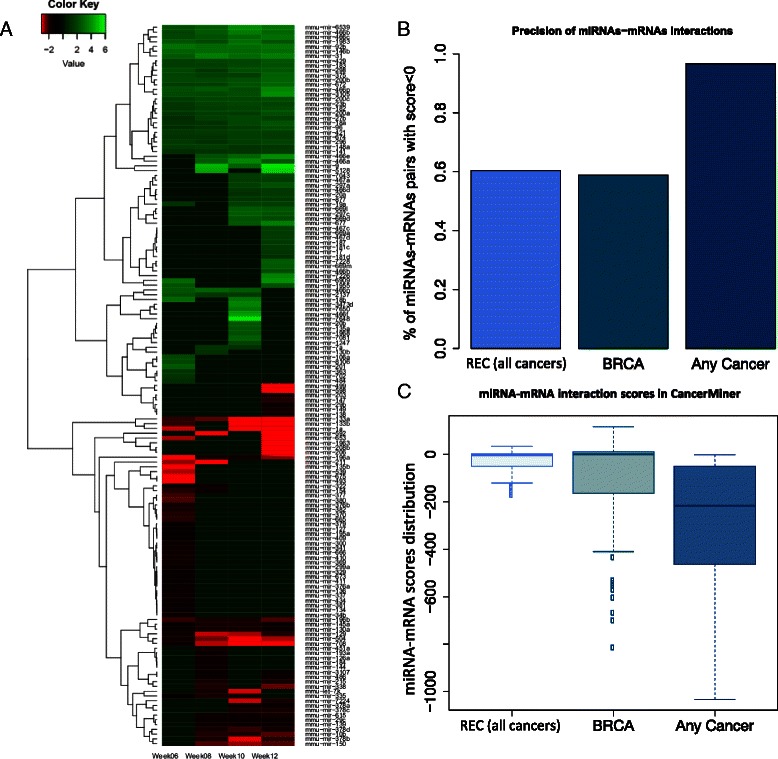
MicroRNA (*MiRNA*) expression analysis and miRNA-messenger RNA (*mRNA*) interaction validation. **a** MiRNA expression profiles during breast cancer (*BRCA*) progression in PyMT mice. The hierarchical clustering of miRNAs was carried out with the Ward method and the Euclidean distances of their expression profiles. The *red dots* indicate the miRNAs already present in another breast cancer study. **b** Comparison of PyMT miRNA-mRNA interactions with their human cancer orthologs. From the CancerMiner database for each human orthologous miRNA-mRNA pair, we calculated the average of recurrent scores for all cancer types and obtained the specific score for breast cancer and the minimum score for any cancer type. The more negative the score, the more reliable the validation of the interaction. Therefore, 0 was used as the threshold to differentiate false and true positives. **c** Score distributions of human orthologs of PyMT miRNA-mRNA interactions in CancerMiner. The negative values indicate the interactions are validated by all three different datasets

By comparing miRNA expression between tumor and normal samples at each of the four time points in our study, we identified 151 differentially expressed miRNAs at one or more time points during breast cancer progression in PyMT mice (Fig. 3a). They can be separated into two groups: 75 miRNAs over-expressed in tumor and the remaining 76 under-expressed. The 10 most upregulated miRNAs at any of the four time points are miR-9, miR-466a/e/f/p, miR-3105, miR-5128, miR-6539, miR-6909, and miR-7648. The 10 most inhibited miRNAs are miR-1a, miR-133a/b, miR-196a, miR-206, miR-208b, miR-211, miR-592, miR-653, and miR-1963. There were 80 differentially expressed miRNAs at two or more time points and, significantly, all of them had consistent directions of differential expression at different time points. This dichotomous differential expression pattern suggests that these miRNAs function as promoters or suppressors in PyMT breast cancer development. Our survey of relevant literature on human and mouse breast cancer revealed that 70 of the 151 differentially expressed miRNAs were shown to be differentially expressed in previous studies (Additional file 1: Supplementary text). Among them, 30 appeared to act as tumor promoters and the other 40 as suppressors. The number of over-expressed miRNAs increases along the four time points. Among over-expressed miRNAs, e.g., miR-31, miR-96, and miR-92b, which are known to be involved in cancer progression, eight miRNAs (miR-466b/c/p, miR-674, miR-672, miR-1983, miR-3105, and miR-6539) have not been shown to play a role in breast cancer progression. We compared the expression profiles of 94 miRNAs differentially expressed in PyMT mice with their corresponding human orthologs in miRCancer [30] and found a large degree of concordance in their expression patterns. When only breast cancer was considered, 77.14 % of the intersecting miRNAs shared the same expression direction (upregulation or downregulation) in cancer in humans and mice. When all cancer types were considered, the concordance rate increased to 89.06 %.

By considering both expression profiles and computational predictions based on sequence complementarity, we identified a total of 10,334 interactions between 178 miRNAs and 1553 mRNAs. In the dense miRNA-mRNA bipartite networks formed by these interactions considering each week independently, miRNAs regulate on average 46 genes and genes are regulated on average by 6 miRNAs. We used a two-step approach - integrated predictions followed by expression-based filtering - to increase the robustness of the bioinformatic prediction of miRNA targets. We compared the resulting miRNAs-mRNA interactions with the ones validated and compiled in CancerMiner [32]. Approximately 60 % of miRNA-mRNA pairs identified in PyMT mice are consistent with those appearing either recurrently in 11 different human cancers or only in human breast cancers. This precision increases to approximately 100 % when only miRNA-mRNA interactions that appear in at least one of the 11 human cancers included in CancerMiner are considered (Fig. 3b).

### Novel miRNAs involved in PyMT breast cancer

Among 151 differentially expressed miRNAs that we identified in breast cancer in PyMT mice, 82 were not previously known to play a role in breast cancer in either humans or mice. Of these novel breast cancer miRNAs, 35 were found to potentially regulate 271 genes based on their sequence complementarity, expression correlation, and expression change direction (Additional file 2: Table S4). Further examination showed that 26 of them could regulate 69 genes that are known to be involved in breast cancer-related pathways and biological processes. We used Gene Ontology (GO) Biological Process and KEGG pathways to annotate these miRNAs based on their potential gene targets. Among them, 22 potentially regulate targets involved in cell and focal adhesion functions [GO:0030155, GO:0007156, KEGG:04510, and KEGG:04514], 10 linked with cell migration [GO:0016477 and GO:0030335], and 7 related to angiogenesis [GO:0001525] or tight junction [KEGG:04530]. We also carried out Interpro domains analysis and found 14 and 10 miRNAs that regulate gene targets with Fibronectin, type III domain (IPR003961) and epidermal growth factor-like domains (IPR006210, IPR000742, and IPR001881), respectively. This indicates a direct relationship of the regulatory activity of these novel miRNAs in cancer processes.

### Functional assessment of differential miRNAs regulation

**Fig. 4:**
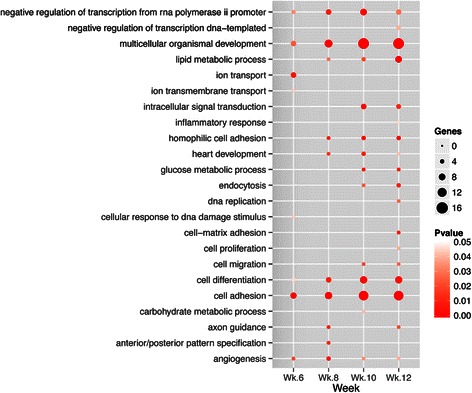
Functional enrichment analysis. The statistically enriched Gene Ontology (GO) terms about biological processes were identified among genes potentially regulated by differentially expressed microRNAs (miRNAs) at each assayed time point. The size of the *dot* indicates the number of genes with the annotation of the term. Its *color* indicates the statistical significance of the enrichment. Its absence indicates the annotation is not enriched at the given time point. Biological terms appear in an alphabetical order in the figure. The full enrichment analysis results are included in the supplementary material (Additional file 2)

As the tumor develops in PyMT mice, more regulated genes become involved in cancer-related biological processes and pathways, including cell adhesion, cell differentiation, multicellular organismal development, tight junction, and cell adhesion molecules (Fig. 4 and Additional file 2: Table S5), which all include both upregulated and downregulated genes. Such increase confirms the active growth related to metastasis, especially in weeks 10 and 12, affecting different aspects of the cancer including cancer metabolism such as lipid and glucose metabolic processes. MiRNAs related to angiogenesis are more active at the first two time points, in agreement with not only the essential role of the blood vessel formation in the early stages of cancer progression but also observations made in previous studies that the transition from dysplasia to adenoma in PyMT mice between 5 and 8 weeks coincides with activation of angiogenesis largely mediated by an influx of macrophages into the tumor tissue [43] in a vascular endothelial growth factor-dependent manner [44]. This finding implies that the early activation of angiogenesis in PyMT mice is probably due to the dysregulation of miRNAs. For example, miR-182 and miR-148a are differentially expressed in weeks 6 and 8, and they regulate *ENPEP*, *EFNB2*, *S1PR1*, and *MMP19* - four genes related to blood vessel morphogenesis or vascular endothelial growth factor (VEGF) signaling.

**Fig. 5:**
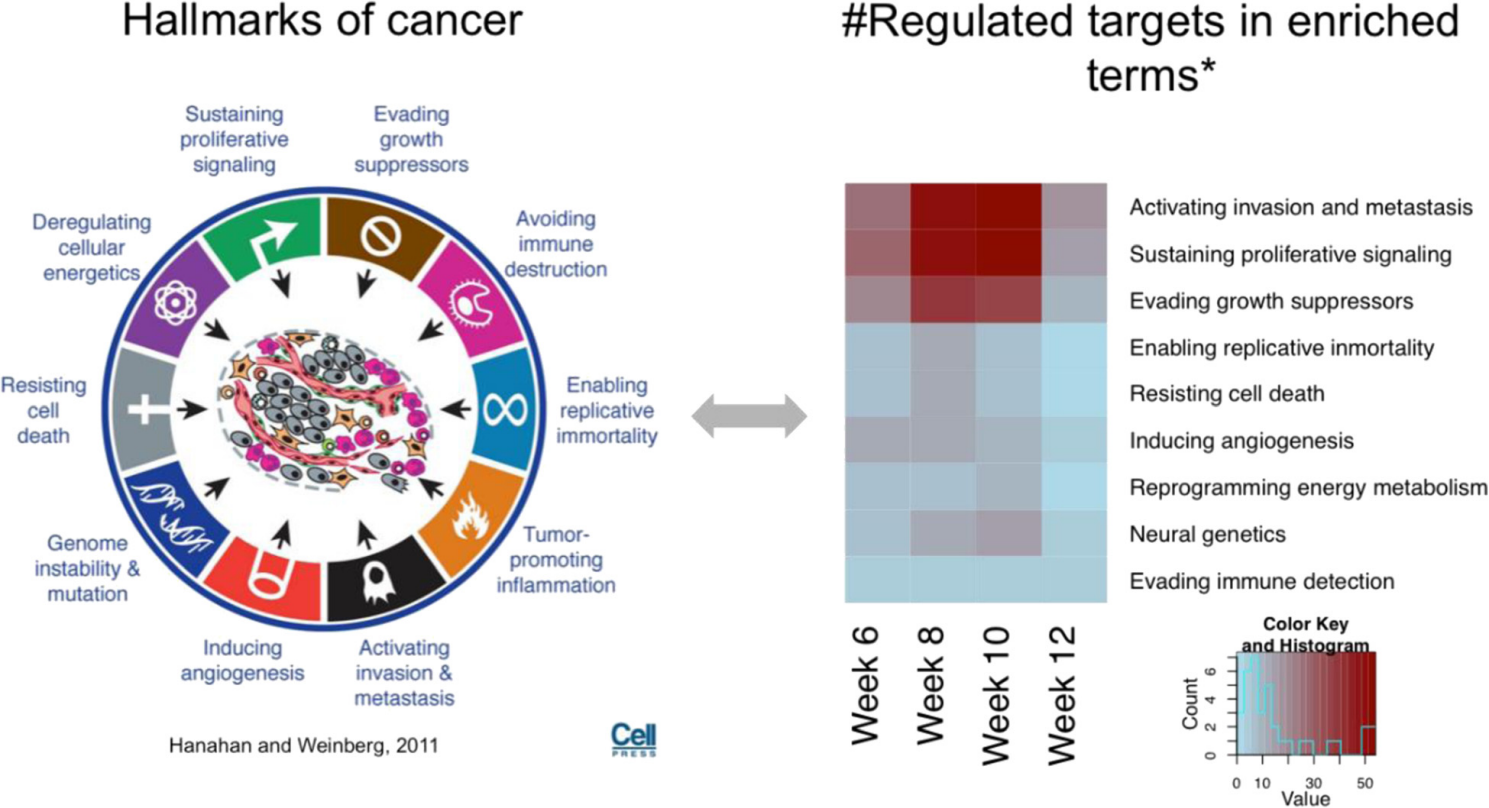
MicroRNA (MiRNA) gene targets and hallmarks of cancer. Enriched functional and pathway terms were combined according to the hallmarks of cancer [38]. The *heatmap* indicates the number of miRNA-regulated genes annotated to each hallmark at each time point. An additional category, 'Neural genetics', was added to combine biological terms related to neural processes and disorders. *Red* indicates high transcriptional activity for the corresponding hallmark and time point. The heatmap suggests that activation invasion and metastasis, sustaining proliferative signaling, and evading growth suppressors are more active, especially in the transition to the carcinoma stages

The comparison between the enriched functional and pathway terms and the proposed hallmarks of cancer [38] revealed two key features of the involvement of miRNAs in breast cancer development (Fig. 5). First, miRNA regulatory activity is focused on three different aspects of cancer: activating invasion and metastasis, sustaining proliferative signaling, and evading growth suppressors. Second, miRNA regulation is more active between weeks 8 and 10, during which the tumor transitions from adenoma to carcinoma. The only exception of cancer hallmarks under miRNA regulation to this time frame is the induction of angiogenesis, which, as shown earlier, is more active in the first 2 weeks.

We identified 18 and 26 miRNAs with negative and positive transition patterns, respectively, along the four time points and analyzed their gene targets separately. The majority of enriched pathways are related to gene targets regulated by miRNAs with higher expression levels, indicating an increased miRNA regulatory activity during cancer progression. The enriched pathways include WNT, hedgehog, and calcium signaling pathways, which are highly related to breast cancer development and cell migration [45, 46].

### Identification and characterization of FRPs

Regulation of any biological processes involves multiple miRNAs and their gene targets. We integrated miRNA target prediction, gene expression profiling, and functional annotation to obtain a global picture of miRNA gene regulatory programs behind breast cancer development and insights into their functional implications. Data integration enabled us to identify FRPs from the mRNA-miRNA bipartite network at each time point and for miRNAs with positive or negative trends in expression along the time points (Additional file 2: Table S6). Some of these programs appear either concurrently along the whole progression or in certain sequential phases (Table 1). Interestingly, 7 out of 12 are especially related to transmembrane processes and the passage of molecules across the cell membrane. We can infer from here the importance of this traffic in phases like activating invasion and metastasis, inducing angiogenesis or proliferation and the special role of miRNA regulation. Some of them, like epidermal growth factor, angiogenesis, cell adhesion molecules, and calcium ion transport are well-known to be relevant to many different aspects of cancer. For example, miR-182, miR-141, miR-18b, miR-200a, miR-17 and, especially, miR-421 appear recurrently along PyMT cancer progression regulating genes involved in ABC transporters (*DNAH8*, *ABCC5*, *ABCA8A*, *ABCB4*, and *ABCA8B*), which are significant in cancer research due to their impact in treatment resistance [47].

**Table 1:** Recurrent microRNA (miRNA) regulatory modules in PyMT breast cancer

Module	Number of genes	Number of miRNAs	Genes regulated during tumor progression
ABC transporters	22	69	*DNAH8 ABCC5 ABCA8A ABCB4 ABCA8B*
GPCR + cell surface receptor	18	63	*ADGRB2 ADGRE5 ADGRG3 ADGRL1 ADGRL4 FZD9 L1CAM*
Fatty acid metabolism	16	57	*ACSL1 ACADL ECH1 ECHS1 HPGD PPARA SNCA*
Cytokine-cytokine receptor + immune response	50	100	*IL1B CX3CR1 PDGFRA GDF5 PDGFRB CXCL15 CXCL1 TNFRSF11A IL7R SBSPON BMP6*
Epidermal growth factor	32	87	*C1RA CCBE1 BMP1 NPNT NRG4 CD93 DLK1 ADGRE5 FAT4 WIF1 LAMA4 ADGRL4 TLL1 ADAM19*
Cell adhesion molecules	41	86	*JAM3 MPZ PTPRM MPZL1 CADM3 NLGN2*
Calcium ion transport	16	56	*ATP2C2 GJA4 CACHD1 TRPM6 CACNA1H RYR2 CYP27B1 CACNA1A RAMP2 JPH2*
Calcium signaling + GPCR, rhodopsin-like domain	40	80	*MYLK NOS1 PDGFRA AGTR1A PTGER3 HRH1 PDGFRB HTR7 PTGER1 HTR4 CACNA1H ADRB3 RYR2 SLC8A2 ADCY3 CACNA1A HTR2A LPAR2 S1PR1 TSPO*
Fibronectin domain	49	101	*CHL1 PHYHIP SPEG EPHA2 MYLK PTPRM MYOM3*
GTP-binding + RAS GTPase	22	68	*RAB36 RAB3D RAB15*
Angiogenesis	18	52	*EPHA2 ANGPT2 JAM3 TNFAIP2 CCBE1 ENPEP FGF1 CYP1B1 EFNB2 S1PR1 TGFA RAMP2 MMP19*
Axon guidance + semaphorin domain	23	76	*SRGAP3 SEMA4G EPHA2 SEMA3A PLXNA1 EFNB1 SEMA3C DPYSL2 NFATC2 RGS3 L1CAM ABLIM2 LRRC4C SLIT3 PLXND1 ITGB3*

After we clustered miRNAs according to their expression profiles (Additional file 2: Table S7), the groups of miRNAs associated with the most gene targets are the ones with significant changes in expression only between weeks 10 and 12, the last two time points. The groups of miRNAs with positive and negative transitions in their expression profiles are composed of 12 and 6 miRNAs, potentially regulating 141 and 49 genes, respectively. We analyzed the molecular functions of these three groups of gene targets (Additional file 2: Table S6). MiRNAs that become active in week 12 are particularly important, associated with protein phosphorylation, regulation of epithelial cell proliferation, calcium and WNT signaling pathways, cytoskeletal regulation by rho GTPase, and transmembrane transports, all of which are linked to metastasis during tumor progression at that stage. On the other hand, miRNAs with only decreased activity in week 12 are associated with transforming growth factor (TGF)-*β* signaling pathway. This is especially interesting given the metastatic role of this pathway in the later phases of breast cancer [48].

### Fatty acid metabolism and breast cancer progression

One hallmark of cancer is the deregulation of cellular energetics. Metabolism of tumorigenic cells is altered to assist their rapid proliferation and growth, invasion, and metastasis [49]. Despite the diverse behavior of different types of cancer, they usually share a similar rewiring of their metabolic pathways [50]. In addition to glycogen metabolism, of which the regulation and implication in cancer cell physiology have been extensively studied [51], fatty acid metabolism is also closely connected to cancer development due to its central role in energy storage, membrane proliferation, and generation of signaling molecules [50]. In our study, the FRP associated with fatty acid metabolism appear especially active in weeks 8, 10, and 12 of PyMT breast cancer progression.

**Fig. 6:**
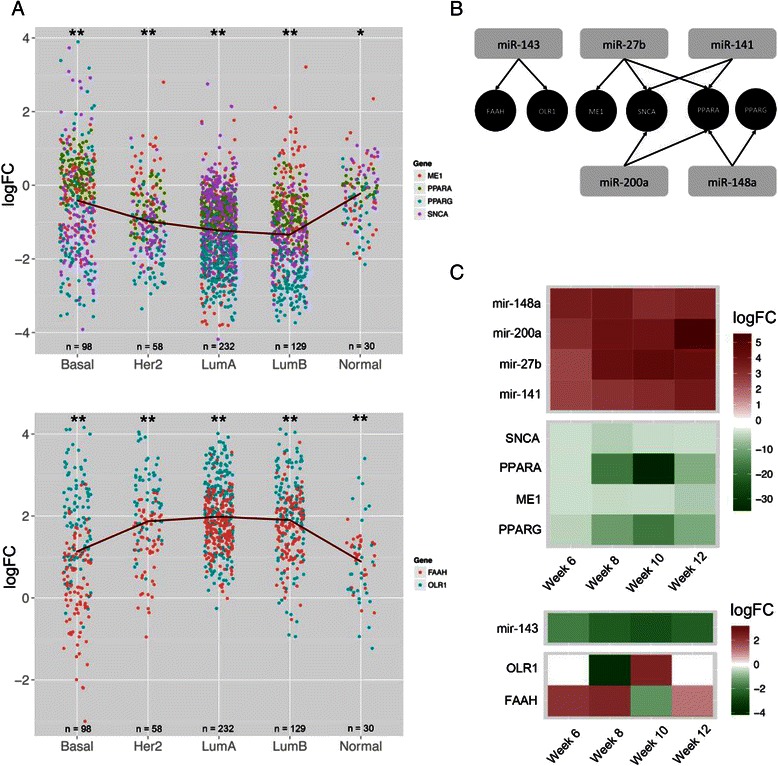
Regulation of fatty acid metabolism. **a** Expression profiles along all breast cancer subtypes in The Cancer Genome Atlas dataset of *OLR1*, *FAAH*, *SNCA*, *ME1*, *PPARA* and *PPARG* genes. *FC* fold change (expression in tumor samples divided by that in normal samples). **b** Recurrent regulatory network of fatty acid metabolism genes (**c**) Expression profiles of fatty acid metabolism genes and miRNA regulators in the PyMT mouse model. *Her2* human epidermal growth factor receptor 2, *LumA* and *LumB* luminal A and luminal B

In total, 31 genes and 67 miRNAs participate in fatty acid metabolism at any of the progression phases. Only six (*FAAH*, *ME1*, *OLR1*, *PPARG*, *PPARA*, and *SNCA*) of them appear to be simultaneously regulated by miRNAs at three different time points. Using the TCGA dataset [52], we studied their expression profiles in all breast cancer subtypes and found that *OLR1* and *FAAH* are consistently over-expressed while *SNCA*, *ME1*, *PPARA*, and *PPARG* are under-expressed in Her2, and luminal A and B subtypes (Fig. 6a). These six genes are also expressed in PyMT mice in different phases of cancer progression or along the whole tumor development (Fig. 6c). MiRNAs persistently regulating these genes in our model are miR-143, miR-27b, miR-141, miR-200a, and miR-148a (Fig. 6b).

It is particularly noteworthy that the expression of *PPARA* and *PPARG* are especially inhibited along the whole cancer progression, especially in week 10. As the peroxisome proliferator-activated receptors, they play key roles in fatty acid oxidation [53]. Although *PPAR* ligands in breast cancer cells remain to be identified, these two genes have been connected to breast cancer and the effects of using them as targets in plausible treatment have been studied [54]. In PyMT mice, miR-200a and miR-148a can regulate their expression, and thus changes in the expression of these miRNAs will probably also drive changes in the expression of both *PPAR* genes. In our study, we also observed the downregulation of *ME1* and *SNCA* transcription in PyMT mice, despite low variability in their expression during tumor development. ME1 is known to play an active role in glutamine metabolism, which produces NADPH, a cofactor important for nucleotide and lipid synthesis [55]; it has not been implicated in breast cancer by past studies. SNCA functions as a fatty acid binding protein and has been identified as a plausible target for early detection or risk assessment of breast cancer [56]. On the other hand, *OLR1* and *FAAH* are over-expressed at different time points during PyMT breast cancer progression. *OLR1* has been characterized as an oncogene in several cell lines, promoting proliferation, migration and inhibition of apoptosis and lipogenesis [57]. *FAAH* has also been reported to be over-expressed in breast cancer cells and studied as a plausible inhibition target for cancer treatment. In our study, sequence analysis showed that both genes contain sequences targetable by miR-143. The co-occurrence of the over-expression of *OLR1* and *FAAH* and the under-expression of miR-143 strongly indicates that both genes could be regulated by this miRNA.

## Conclusions

In this study we profiled the expression of miRNAs during breast cancer progression in the PyMT mouse model. This model closely resembles ER-positive luminal B breast cancer, a subtype of breast cancer that traditionally is poorly studied in humans. Instead of a particular cancer state, we considered the whole disease progression, sampling four time points to represent hyperplasia, adenoma, early carcinoma, and late carcinoma phases. By integrating miRNA and mRNA expression profiles, we identified 151 differentially expressed miRNAs and their target genes involved in the regulatory activities of cancer biology, with a strict dual nature of either upregulation or downregulation during the whole disease progression. From the comparison with miRCancer as an assessment of concordance with other miRNA studies as a whole, we observed high-level agreement in miRNA expression between breast cancer in PyMT mice and human cancers reported in previous studies. The expression profiles of several key miRNAs from PyMT mice in our study matched those from previously obtained ER-positive cancers, confirming the luminal B subtype status of the PyMT mouse model, while different expression profiles of signature miRNAs for the triple-negative subtype indicated their divergence. Using this model, we identified 82 novel breast cancer-related miRNAs, 35 of which can potentially regulate 271 protein-coding genes based on sequence complementarity and expression profiles. After a comprehensive study, we found that a subset of 26 novel miRNAs potentially regulate 69 genes that are implicated in biological processes related to cancer biology, including cell and focal adhesion functions, cell migration, and angiogenesis. During breast cancer progression in PyMT mice, genes regulated by significantly over-expressed miRNAs participate in cell adhesion or cell differentiation, biological processes related to the cancer progression and metastasis. Collated with cancer hallmarks, our study showed that genes targeted by miRNAs with perturbed expression in breast cancer in PyMT mice are involved in activating invasion and metastasis, sustaining proliferative signaling, and evading growth suppressors during the transition from adenoma to carcinoma. Finally, applying a novel clustering method to an annotated miRNA-mRNA regulatory network, we identified 84 FRPs, all of which are involved in cancer-related biological processes, including metabolism, endocytosis, transmembrane transport, and the cellular immune response.

## Abbreviations

ER, estrogen receptor; FRP, functional regulatory program; HER2, human epidermal growth factor receptor 2; KEGG, Kyoto Encyclopedia of Genes and Genomes; MB, maximal biclique; miRNA, microRNA; mRNA, messenger RNA; PR, progesterone receptor; PyMT, polyoma middle T antigen; RNA-seq, RNA sequencing; TCGA, The Cancer Genome Atlas

## Additional files

Additional file 1: Supplementary text with references and supplementary figures. (DOCX 3756 kb)
Additional file 2: Supplementary tables. (XLSX 1081 kb)
