# Supplementary material for: MicroRNA expression and gene regulation drive breast cancer progression and metastasis in PyMT mice
Source: Breast Cancer Res. 2016 Jul 22;18:75. doi: 10.1186/s13058-016-0735-z (PMC4957901; doi:10.1186/s13058-016-0735-z)
Supplement: Additional file 1: — Supplementary text with references and supplementary figures. (DOCX 3756 kb) [file 13058_2016_735_MOESM1_ESM.docx]

# supplementary text

## Expression of known breast cancer-related miRNAs in PyMT mice

Among miRNAs linked to breast cancer, many of them have been extensively studied to illustrate their functional roles in the disease. To investigate how these miRNAs are related to the tumor development in PyMT mice, we examined their expression patterns in our small RNA-seq time-series data.

MiR-17~92 are one of the best-studied miRNA clusters and have been shown to play a role in tumorigenesis [[1](#_ENREF_1)]. *PTEN* is one of the first validated gene targets of this cluster [[2](#_ENREF_2)]. During breast cancer progression in PyMT mice, our study shows that various members of the miR-17~92 cluster were statistically up-regulated at different time points, especially in weeks 10 and 12. Moreover, *PTEN* was down-regulated at the last three time points. The down-regulation of *PTEN* further activates the EMT process, one of the crucial steps during metastasis [[3](#_ENREF_3)] [[4](#_ENREF_4)]. In agreement with our results despite different breast cancer subtypes, deep sequencing of triple negative breast cancer samples revealed a threefold increase of miR-17~92 levels [[5](#_ENREF_5)].

In ER-positive breast cancer, it was shown that miR-18a/b directly bind the 3′ UTR of *ERα* and thus suppress its expression [[6](#_ENREF_6)]. In addition, miR-17 and miR-20 are overexpressed in metastatic breast cancer and have been shown to directly suppress *IL*-8 [[7](#_ENREF_7)]. Both *ERα* and *IL*-8 were strongly down-regulated along the four times points in PyMT mice. MiR-96 and miR-143 are two other miRNAs important in cancer. MiR-96 promotes tumor proliferation and invasion by targeting RECK in breast cancer [[8](#_ENREF_8)], while miR-143 suppresses the expression of Hexokinase 2 (HK2) [[9](#_ENREF_9)], which is required for tumor initiation and maintenance in mouse cancer models [[10](#_ENREF_10)]. The expression of these two miRNAs and their gene targets in PyMT mice are in agreement with previously published results. Previous studies have shown that the miR-200 family (miR-200a/b/c, mir-141, and mir-429) suppresses EMT in metastasis, mediated mainly through the regulation of E-Cadherin (CDH1) transcriptional repressors ZEB1 and ZEB2 [[11](#_ENREF_11)] [[12](#_ENREF_12)]. This miRNA family was up-regulated, being ZEB1 strongly down-regulated and, therefore, CDH1 highly up-regulated. This expression pattern suggests a suppression of EMT, in agreement with the conclusion made by a previous that EMT is absent in luminal-like breast cancer [[13](#_ENREF_13)]. Another plausible explanation is that EMT could happen by the aforementioned regulation of PTEN.

MiR-10b is known to play a pivotal role in the development process [[14](#_ENREF_14)]. Together with miR-148a, -150, -199a, and -486, it is only expressed in normal mouse mammary epithelium and not in tumors, suggesting that they may have tumor suppressor activities [[15](#_ENREF_15)]. In the same study, miR-21, one of the most important miRNAs associated with cell migration and invasion in breast cancer cells [[16](#_ENREF_16)] [[3](#_ENREF_3)], was not found differentially expressed, indicating the different and complex regulatory networks associated with miRNAs in different kind of cells. In the case of miR-21, its metastatic activity is highly associated with the regulation of PTEN, also targeted by mir-17~92 cluster.

In our study, several miRNAs showed transcriptional activities different from previous reports. Studies have shown that miR-31 suppresses cancer metastasis in both human and mouse and is specifically attenuated in aggressive breast cancer cells through the regulation of pro-metastatic oncogenes like WAVE3 [[17](#_ENREF_17)], RhoA, and Radixin [[18](#_ENREF_18)]. Its activity has been associated mainly in the triple-negative breast cancer [[18](#_ENREF_18)]. Probably due to the differences between breast cancer subtypes, in our study miR-31 was up-regulated in PyMT breast cancer at all four time points. Indeed, a previous study showed very different expression profiles of miR-31 in the luminal and basal breast caner cells. While the mature miR-31 is highly expressed in MCF7, SKBr3, and T47D cell lines of the luminal breast cancer subtypes, to which breast cancer in PyMT mice belong, its expression is significantly reduced in BT549, MDA-MB-231, and MDA-MB-453S cell lines of the triple-negative breast cancer subtype [[19](#_ENREF_19)]. MiR-146 was previously reported to suppress migration and invasion in human breast cancer [[19](#_ENREF_19)], more related with triple negative breast cancers [[20](#_ENREF_20)]. In our study of mouse luminal-like breast cancer, miR-146b was up-regulated at all four time points.

We also examined in PyMT mice the expression of miR-9, another cancer-related miRNA, which activates the JAK-STAT pathway. Transported in microvesicles to endothelial cells, it targets SOCS5, causing endothelial cell migration and tumor angiogenesis in human [[21](#_ENREF_21)]. It has been observed that overexpression of miR-9 and down-regulation of miR-10b lead to EMT. This happens through the down-regulation of CDH1, which causes the release of beta-catenin in the nucleus of cells and the expression of VEGF [[22](#_ENREF_22)]. Despite such observations, the involvement of miR-9 in breast cancer is not clear. In PyMT mice miR-9 was overexpressed at the last three time points, while SOCS5 was not differentially down-regulated. Moreover, as previously stated, CDH1 was up-regulated in our study, probably mediated by the miR-200 family. Considering the whole expression transitions at the four time points together, we found 17 potential gene targets of miR-9. Functional analysis of those genes with annotations by GO Biological Process and Interpro Domains identified the enrichment of multiple gene annotation terms. Enriched among important miR-9 gene targets such as Kcna2, Kcnq5, and Slc24a2, potassium ion channels and trans-membrane transport have been implicated in angiogenesis and cell migration, processes important to cancer pathophysiology [[23](#_ENREF_23)]. We hypothesis that miR-9 is involved in the regulation of mechanisms related to transmembrane transport by potassium channels to promote metastasis of the cancer.

# References

1. Mogilyansky E, Rigoutsos I: **The miR-17/92 cluster: a comprehensive update on its genomics, genetics, functions and increasingly important and numerous roles in health and disease**. *Cell death and differentiation* 2013, **20**(12):1603-1614.

2. Ventura A, Young AG, Winslow MM, Lintault L, Meissner A, Erkeland SJ, Newman J, Bronson RT, Crowley D, Stone JR *et al*: **Targeted deletion reveals essential and overlapping functions of the miR-17 through 92 family of miRNA clusters**. *Cell* 2008, **132**(5):875-886.

3. Han M, Liu M, Wang Y, Chen X, Xu J, Sun Y, Zhao L, Qu H, Fan Y, Wu C: **Antagonism of miR-21 reverses epithelial-mesenchymal transition and cancer stem cell phenotype through AKT/ERK1/2 inactivation by targeting PTEN**. *PloS one* 2012, **7**(6):e39520.

4. Jung CJ, Iyengar S, Blahnik KR, Jiang JX, Tahimic C, Torok NJ, de vere White RW, Farnham PJ, Zern M: **Human ESC self-renewal promoting microRNAs induce epithelial-mesenchymal transition in hepatocytes by controlling the PTEN and TGFbeta tumor suppressor signaling pathways**. *Molecular cancer research : MCR* 2012, **10**(7):979-991.

5. Farazi TA, Horlings HM, Ten Hoeve JJ, Mihailovic A, Halfwerk H, Morozov P, Brown M, Hafner M, Reyal F, van Kouwenhove M *et al*: **MicroRNA sequence and expression analysis in breast tumors by deep sequencing**. *Cancer research* 2011, **71**(13):4443-4453.

6. Leivonen SK, Makela R, Ostling P, Kohonen P, Haapa-Paananen S, Kleivi K, Enerly E, Aakula A, Hellstrom K, Sahlberg N *et al*: **Protein lysate microarray analysis to identify microRNAs regulating estrogen receptor signaling in breast cancer cell lines**. *Oncogene* 2009, **28**(44):3926-3936.

7. Yu Z, Willmarth NE, Zhou J, Katiyar S, Wang M, Liu Y, McCue PA, Quong AA, Lisanti MP, Pestell RG: **microRNA 17/20 inhibits cellular invasion and tumor metastasis in breast cancer by heterotypic signaling**. *Proceedings of the National Academy of Sciences of the United States of America* 2010, **107**(18):8231-8236.

8. Zhang J, Kong X, Li J, Luo Q, Li X, Shen L, Chen L, Fang L: **miR-96 promotes tumor proliferation and invasion by targeting RECK in breast cancer**. *Oncology reports* 2014, **31**(3):1357-1363.

9. Jiang S, Zhang LF, Zhang HW, Hu S, Lu MH, Liang S, Li B, Li Y, Li D, Wang ED *et al*: **A novel miR-155/miR-143 cascade controls glycolysis by regulating hexokinase 2 in breast cancer cells**. *The EMBO journal* 2012, **31**(8):1985-1998.

10. Patra KC, Wang Q, Bhaskar PT, Miller L, Wang Z, Wheaton W, Chandel N, Laakso M, Muller WJ, Allen EL *et al*: **Hexokinase 2 is required for tumor initiation and maintenance and its systemic deletion is therapeutic in mouse models of cancer**. *Cancer cell* 2013, **24**(2):213-228.

11. Li X, Roslan S, Johnstone CN, Wright JA, Bracken CP, Anderson M, Bert AG, Selth LA, Anderson RL, Goodall GJ *et al*: **MiR-200 can repress breast cancer metastasis through ZEB1-independent but moesin-dependent pathways**. *Oncogene* 2014, **33**(31):4077-4088.

12. Park SM, Gaur AB, Lengyel E, Peter ME: **The miR-200 family determines the epithelial phenotype of cancer cells by targeting the E-cadherin repressors ZEB1 and ZEB2**. *Genes & development* 2008, **22**(7):894-907.

13. Trimboli AJ, Fukino K, de Bruin A, Wei G, Shen L, Tanner SM, Creasap N, Rosol TJ, Robinson ML, Eng C *et al*: **Direct evidence for epithelial-mesenchymal transitions in breast cancer**. *Cancer research* 2008, **68**(3):937-945.

14. Lund AH: **miR-10 in development and cancer**. *Cell death and differentiation* 2010, **17**(2):209-214.

15. Zhu M, Yi M, Kim CH, Deng C, Li Y, Medina D, Stephens RM, Green JE: **Integrated miRNA and mRNA expression profiling of mouse mammary tumor models identifies miRNA signatures associated with mammary tumor lineage**. *Genome biology* 2011, **12**(8):R77.

16. Han M, Liu M, Wang Y, Mo Z, Bi X, Liu Z, Fan Y, Chen X, Wu C: **Re-expression of miR-21 contributes to migration and invasion by inducing epithelial-mesenchymal transition consistent with cancer stem cell characteristics in MCF-7 cells**. *Molecular and cellular biochemistry* 2012, **363**(1-2):427-436.

17. Sossey-Alaoui K, Downs-Kelly E, Das M, Izem L, Tubbs R, Plow EF: **WAVE3, an actin remodeling protein, is regulated by the metastasis suppressor microRNA, miR-31, during the invasion-metastasis cascade**. *International journal of cancer Journal international du cancer* 2011, **129**(6):1331-1343.

18. Valastyan S, Reinhardt F, Benaich N, Calogrias D, Szasz AM, Wang ZC, Brock JE, Richardson AL, Weinberg RA: **A pleiotropically acting microRNA, miR-31, inhibits breast cancer metastasis**. *Cell* 2009, **137**(6):1032-1046.

19. Augoff K, McCue B, Plow EF, Sossey-Alaoui K: **miR-31 and its host gene lncRNA LOC554202 are regulated by promoter hypermethylation in triple-negative breast cancer**. *Molecular cancer* 2012, **11**:5.

20. Garcia AI, Buisson M, Bertrand P, Rimokh R, Rouleau E, Lopez BS, Lidereau R, Mikaelian I, Mazoyer S: **Down-regulation of BRCA1 expression by miR-146a and miR-146b-5p in triple negative sporadic breast cancers**. *EMBO molecular medicine* 2011, **3**(5):279-290.

21. Zhuang G, Wu X, Jiang Z, Kasman I, Yao J, Guan Y, Oeh J, Modrusan Z, Bais C, Sampath D *et al*: **Tumour-secreted miR-9 promotes endothelial cell migration and angiogenesis by activating the JAK-STAT pathway**. *The EMBO journal* 2012, **31**(17):3513-3523.

22. Ma L, Young J, Prabhala H, Pan E, Mestdagh P, Muth D, Teruya-Feldstein J, Reinhardt F, Onder TT, Valastyan S *et al*: **miR-9, a MYC/MYCN-activated microRNA, regulates E-cadherin and cancer metastasis**. *Nature cell biology* 2010, **12**(3):247-256.

23. Pardo LA, Stuhmer W: **The roles of K(+) channels in cancer**. *Nature reviews Cancer* 2014, **14**(1):39-48.

# supplementary figures

**Supplementary Figure 1.** H&E Staining of tumor samples from PyMT mice at 6, 8, 10, 12 weeks of age


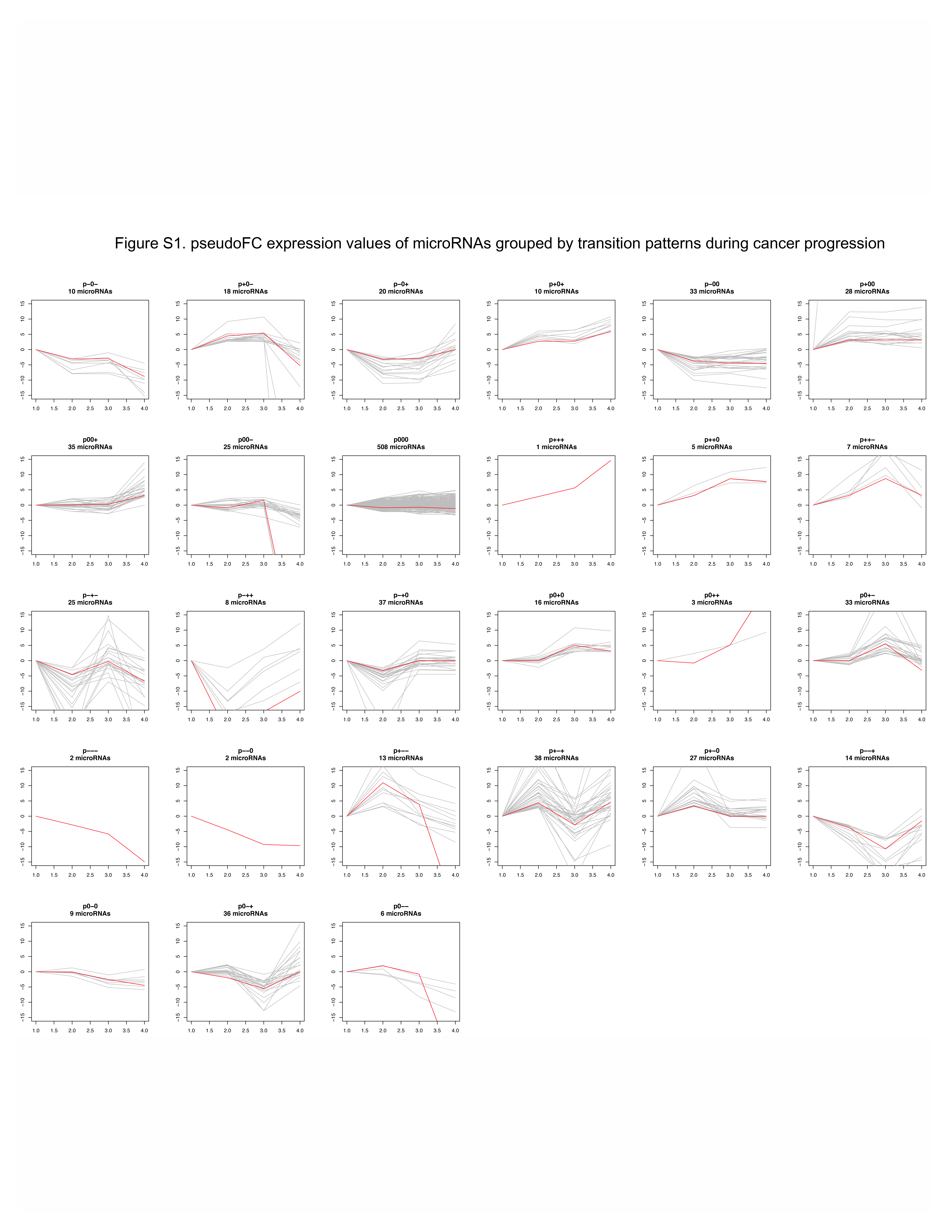


**Supplementary Figure 2.** Pseudo-FC expression values of miRNAs grouped by transition patterns during tumor progression.
